# Spatiotemporal Labeling of Melanocytes in Mice

**DOI:** 10.3390/ijms19051469

**Published:** 2018-05-15

**Authors:** Sarah Preston, Shweta Aras, M. Raza Zaidi

**Affiliations:** Fels Institute for Cancer Research & Molecular Biology, Lewis Katz School of Medicine at Temple University, Philadelphia, PA 19140, USA; sarah.preston@temple.edu (S.P.); shweta.aras@temple.edu (S.A.)

**Keywords:** melanoma, melanocyte, genetically engineered mouse models, iDct-GFP, FUCCI

## Abstract

Melanocytes are pigment producing cells in the skin that give rise to cutaneous malignant melanoma, which is a highly aggressive and the deadliest form of skin cancer. Studying melanocytes in vivo is often difficult due to their small proportion in the skin and the lack of specific cell surface markers. Several genetically-engineered mouse models (GEMMs) have been created to specifically label the melanocyte compartment. These models give both spatial and temporal control over the expression of a cellular ‘beacon’ that has an added benefit of inducible expression that can be activated on demand. Two powerful models that are discussed in this review include the melanocyte-specific, tetracycline-inducible green fluorescent protein expression system (iDct-GFP), and the fluorescent ubiquitination-based cell cycle indicator (FUCCI) model that allows for the monitoring of the cell-cycle. These two systems are powerful tools in studying melanocyte and melanoma biology. We discuss their current uses and how they could be employed to help answer unresolved questions in the fields of melanocyte and melanoma biology.

## 1. Introduction

Melanocytes are the pigment-producing cells that are primarily located in skin, but are also found in retinal pigment epithelium of eyes, inner ear, and some mucosal surfaces. They produce melanin pigment in specialized intracellular compartments, called melanosomes, which are transferred to neighboring keratinocytes. Melanin production in skin is important for protecting cutaneous cellular nuclei from harmful ultraviolet radiation (UVR), which is the most ubiquitous known carcinogen. Exposure to UVR has been associated with benign nevi, melanocytic lesions, and malignant melanomas [1,2]. It is necessary to fully understand the biology of melanocytes and their response to UVR exposure as well as the indirect consequences of UVR on melanocytes by promoting changes in the skin microenvironment. The ideal setting to study the biology and the function of melanocytes is within its natural primary location of residence where it is exposed to the natural tissue architecture and the cellular microenvironment. Studying melanocytes in vivo has been difficult because they make up ~1% of the cellular milieu within the skin and they do not harbor reliable specific cell-surface markers that could be used to isolate them in any appreciable purity. An effective strategy in overcoming these challenges is the development of transgenic mouse models to “label” melanocytes using lineage-specific gene promoters through spatiotemporal gene regulation of a reporter construct. 

## 2. Labeling the Melanocyte Compartment in Mice 

There is a long history in studying and tracking the orgin of melanoblast precursors from the neural crest during embryogenesis and development [3]. One of the first models that was used to label the melanocytic cell lineage was the Dct-lacZ transgenic mouse. In 1997, MacKenzie et al. developed a transgenic mouse that used the melanocyte-specific dopochrome tautomerase (*Dct*) gene promoter to drive the expression of the Lac operon gene LacZ. DCT catalyzes the conversion of DOPAchrome to 5,6-dihydroxyindole 2-carboxylic acid (DHICA) in the melanin synthesis pathway [4,5]. This gene is expressed in melanocyte precursors, melanoblasts, as well as in differentiated melanocytes [1]. The *Dct* promoter was used in the Dct-lacZ model to drive the expression of a reporter enzyme, beta-galactosidase. This reporter enzyme cleaves its chromogenic substrate X-gal to label cells blue in visible light [5]. This model has been useful in identifying the existence and the location of melanocyte stem cells, and the molecular defects that affect melanocyte development [5,6,7]. However, further utility of this model was limited because it lacks conditionally inducible labeling and could not be used to isolate a pure population of melanocytes. 

## 3. Melanocyte-Specific Labeling in Mouse Models

In addition to the seminal Dct-lacZ model that is described above, there has been an active development in mouse models that use reporters to label the melanocytic cell lineage. Over the years, the following conditional systems have been described: (1) a transgene using the *Dct* promoter to drive expression of a yellow fluorescent protein (YFP) variant [8]; (2) a transgenic system that expresses green fluorescent protein (GFP) upon Cre-mediated excision under the control of Tyrosinase (*Tyr*) promoter [9,10]; and, (3) a similar system that expresses YFP upon Cre-mediated excision [11,12,13]. These systems rely on the *Tyr* or *Dct* promoter to drive the expression of the reporter gene. *Tyr* and *Dct* genes encode for enzymes that are involved in melanin synthesis and are faithful markers of melanoblasts and melanocytes [1,14,15]. A key transcription factor for melanocyte development is the paired box transcription factor *Pax3* [16,17,18]. Expression of a transgenic *Pax3^GFP^* allele encodes GFP in exon 1 of *Pax3* and is an alternative marker for pigmented cells at every developmental stage [19]. With this promoter, there is an additional population of Schwann cell precursors that are separate from the melanocyte lineage, which are also labeled [19]. While these models label pigment cells specifically, they lack the desirable feature of inducible expression. 

## 4. Inducible Marker Expression in Melanocytes

Purification of melanocytes after in vivo manipulation is advantageous for the study of these cells at the cellular and molecular levels. Using expression of a fluorescent protein under a lineage-specific promoter system allows for a pure population of melanocytes to be isolated by fluorescence-activated cell sorting (FACS) [20]. Inducible expression creates a powerful model where timing of the cellular label can be controlled to avoid potential toxicity from chronic expression. For studies involving UVR, it is especially important to induce the expression of fluorescent markers, especially GFP, after irradiation, because GFP can absorb UVR and it may interfere with the physiological response by reducing the amount of effective UVR dose received by the target cells. A popular method to induce-gene expression uses the reverse tetracycline-controlled transactivator gene (rtTA), which will only activate genes under the control of a tetracycline-responsive element (TRE) in the presence of tetracycline or its analog doxycycline. A more stable version of the transactivator, rtTA2s-M2, is preferred to the original since it requires a lower concentration of doxycycline and causes minimal to no leaky background expression in the absence of doxycycline [21]. Using a lineage–specific promoter (e.g., *Dct*) to drive expression of rtTA2s-M2 creates a system that is both targeted and inducible for the melanocyte compartment for virtually any gene under the control of the TRE promoter. The Dct-rtTA transgene has been used to drive expression of both β-gal and GFP reporter genes [20,22]. Another transgenic allele uses *Tyr* promoter to drive the expression of an inducible Cre activity (*Tyr::CreER^T2^*) after tamoxifen treatment to induce GFP or tdTomato expression after Cre-mediated transgene excision [23,24]. This model has since been used to define the role of melanocyte stem cells as melanoma cells of origin antagonized by UVB radiation [25]. Since the expression pattern using this system has not been fully characterized at all of the developmental stages, we will begin our discussion on the use of the iDct-GFP mouse model. 

## 5. The iDct-GFP Mouse Model

**Figure 1:**
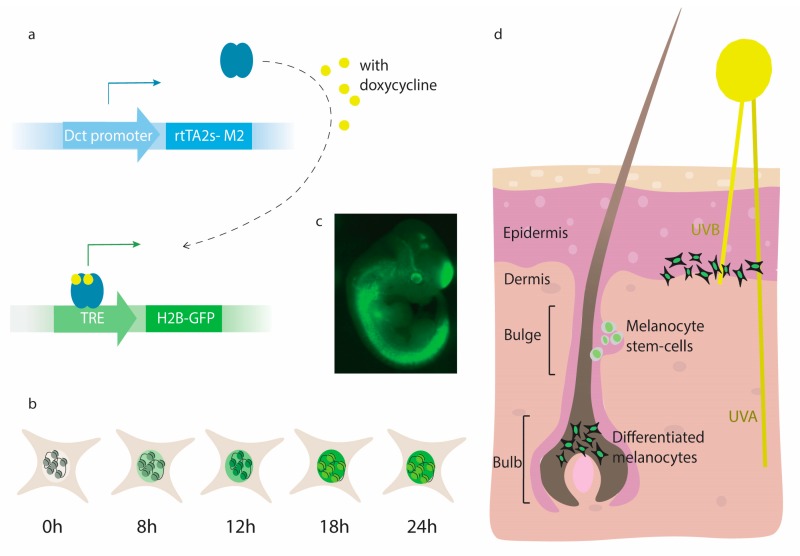
(**A**) The tetracycline-inducible green fluorescent protein (iDct-GFP) transgenic mouse contains two alleles, one uses the Dct promoter to drive the transcription (solid arrow) of rtTA2s-M2 and a second that is doxycycline inducible using a TRE promoted to drive expression of H2B-GFP; (**B**) Peak expression of H2B-GFP occurs at 18 h after administration of doxycycline. It remains stable in the cells and is affected by low rates of protein turn-over and cellular proliferation; (**C**) Expression of GFP-H2B in a mouse embyro (day 11.5) correlates with early Dct expression; (**D**) The hair follicle contains both melanocyte stem-cells located in the bulge region and differentiated melanocytes that are found in the bulb. ultraviolet radiation (UVR) is comprised of UVB wavebands that can only reach just beyond the epidermis and the UVA waveband that can reach to the deep dermis. After UVR exposure, activated melanocytes migrate to the dermal-epidermal junction and differentiate into pigment producing cells.

The iDct-GFP mouse exhibits melanocyte-specific, inducible expression of a GFP fusion protein [20]. It is a compound transgenic mouse that contains the Dct-rtTA transgene to drive the expression of rtTA2s-M2 under the control of the Dct promoter, and a TRE-H2B-GFP transgene to induce the expression of the histone H2B-GFP fusion protein (Figure 1A,B) [26]. These mice have been fully characterized for GFP expression in melanocytic cells at all stages of development, including embryonic, neonatal, and adult [20,27,28]. At embryonic day 9.5 (E9.5), melanocyte precursors, melanoblasts, appear in the neural crest and harbor upregulated melanoblast-specific markers (Mitf, Tyr, Dct, Kit) around E10.5 (Figure 1C) [29]. From the neural crest, melanoblasts migrate via the dorsolateral route to the dermal-epidermal junction and to the hair follicles as they begin to differentiate [30,31]. Eventually, only melanocytes in the bulb, outer root sheath and bulge regions of the hair follicle persist after neonatal development in wild-type mice (Figure 1D) [32]. This is seen in the iDct-GFP mice, which show a GFP pattern in all developmental stages, as consistent with known melanocyte locations and Dct expression. The bright GFP label enables FACS-mediated sorting of melanocytes at early embryonic stages and in adult mice, making this model a powerful tool to study melanocyte development and related diseases. 

The initial use of the iDct-GFP model studied the melanocyte response to UVR, while residing in their natural physiological microenvironment. UVR is a complete carcinogen, which was shown to both initiate and promote tumorigenesis; however, the underlying molecular mechanisms that are promoted by UVR are largely unknown. UVR exposure in neonatal iDct-GFP mice showed the UVB, but not the UVA, waveband, to be largely responsible for eliciting a melanocyte response (“activation”), resulting in both the proliferation and upward migration towards the epidermis of GFP labeled cells [27]. There is growing evidence for waveband-specific tumorigenic mechanisms; for example, it has been previously shown that UVB but not UVA is tumorigenic in the albino HGF/SF-Tg mouse model [1,33]. Further transcriptomic analysis of UVB-activated melanocytes by microarray showed an early stress response that included an interferon-γ gene expression signature [27]. A subset of macrophages infiltrated the skin in response to UVB exposure and was identified as the source of interferon-γ. These UVB-recruited macrophages enhanced tumor growth when they were transplanted with melanoma cells in syngeneic mice. The secretion of interferon-γ from these macrophages was the active agent that promoted tumor growth, and this effect was abrogated by blockade with an anti-interferon-γ antibody within the host mice. The role of these interferon-γ response genes in melanocyte biology remains to be elucidated, but it is interesting that many already have known roles in immune evasion and suppression, including but not limited to cytotoxic T-lymphocyte antigen 4 (CTLA4). CTLA4 is an immune-checkpoint molecule that auto-attenuates effector T-cell function to enforce tolerance to cognate antigens. We have shown that human melanomas that express CTLA4 and other interferon-γ response genes had significant durable responses to anti-CTLA4 immunotherapy, ipilimumab [34]. This implicates a role for targeting CTLA4 on melanoma cells in mediating the effects of anti-CTLA4 immunotherapy. This interferon-γ response seems to be an important survival signal for melanocytes after UV irradiation and may function in an immunoediting paradigm that allows for the central tolerance of melanocytic antigens. This initial in vivo analysis of melanocyte response to UVR has many implications for not only melanocyte biology but also melanoma biology and clinical therapeutics. Future studies using the iDct-GFP mouse in conjunction with models of melanoma should yield meaningful insights of melanoma etiology. 

## 6. The Tyr-Cre LSL-tdTomato Mouse Model

While the iDct*-GFP* mouse is a reliable and effective model to spatially and temporally control expression within the melanocyte compartment, its control is limited to gene constructs with TRE promoters. Cumbersome breeding is required to use this model with the expression of many melanoma oncogenes that rely on Cre-based excision. For these purposes, *Tyr::CreER^T2^* is a useful tool in manipulating the melanocyte compartment and the use of *Tyr::CreER^T2^* in melanoma mouse models has been extensively reviewed [28]. The *Tyr::CreER^T2^* contains a 3.6 kb enhancer and 5.5 kb promoter sequence of the mouse *Tyr* locus, which drives the expression of Cre recombinase [24]. Cre recombinase recognizes a 34 bp sequence, termed LoxP site, and it recombines to excise the DNA sequence that is flanked by LoxP sites (floxed) [35]. When used in combination with the *LSL-tdTomato* transgene, it labels melanocytes following tamoxifen administration to excise the Lox-Stop-Lox (LSL) cassette and allows the full expression of *tdTomato* [36]. This *Tyr::CreER^T2^* construct has been used to simultaneously label and drive the expression of melanomagenic programs within the melanocyte compartment to study if the melanoma cell of origin arises from melanocyte stem cells (MSCs) or more differentiated pigment-producing melanocytes, a question still outstanding in the field. MSCs, and adult stem-cells in general, have been proposed as cancer cells of origin because of their long life span, immune privilege, and self-renewing potential [37]. Melanomas also display phenotypic heterogeneity, which could be explained if they arose from a cell-type with multi-potent capability. 

The fate of the follicular MSCs to differentiate, proliferate, and survive is closely coupled with the hair cycle [38]. The dorsal skin in mice undergoes frequent hair cycles where the hair follicle and the MSCs are activated during the anagen phase when the hair follicle regenerates. The hair follicle shrinks in the catagen phase and undergoes a period of rest during the intermittent telogen phase where it enters a quiescent state. MSCs are kept in a quiescent state in the bulge until the beginning of anagen where they begin to proliferate and give rise to transient amplifying cells that differentiate to replenish the pigment producing melanocyte population that is located in the lower bulb region. The melanocytes that were located in the bulb die by apoptosis during the catagen phase of the hair follicle [39]. One of the challenges in studying MSCs is distinguishing them from differentiated melanocytes, as no specific markers have been identified. MSCs are characterized by their location in the bulge area, absence of melanin pigment, and the expression of late-differentiation markers, such as tyrosinase-related protein 1 (Tyrp1). While *Tyr* is not commonly considered to be expressed in MSCs, the *Tyr::CreER^T2^* transgene has been shown to be expressed in the MSCs that are residing in the hair follicle bulge region and all of their progeny [24,40]. When crossed with the iDct-GFP mouse, GFP positive cells driven by the Dct promoter overlapped with the *tdTomato*-positive labeling driven by Tyr promoter [25]. Immunostaining for Tyrp1, which is a late maker of differentiated melanocytes, did not label these GFP or *tdTomato* positive cells, indicating that Tyr is active in MSCs [25]. SRY-box 10 (SOX10) is an important transcription factor whose loss of function leads to hair graying. Genetic ablation of *Sox10* by *Tyr::CreER^T2^* led to a deficiency in maintaining the MSC population and their ability to replenish the differentiated melanocytes of the bulb during anagen, illustrating the ability of *Tyr::CreER^T2^* to target the MSC population [41]. 

To investigate the role of MSCs as cancer cells of origin and the cell-extrinsic factors that are required for melanoma development, the *Tyr::CreER^T2^*; *LSL-tdTomato* mice can be crossed with a conditional *Braf^CA/+^* and *Pten^lox/lox^* mouse model. The *Braf^CA/+^* mouse conditionally expresses the constitutively active mutant v-Raf murine sarcoma viral oncogene homolog B (*Braf^V600E^*), which is the most common mutation that is found in human melanomas and it often co-occurs with the loss of the tumor suppressor gene phosphatase and tensin homolog *Pten* [42]. *Braf^V600E^* alone gives rise to highly proliferative melanocytes that succumb to oncogene-induced senescence, but, in combination with *Pten* loss melanomas, develop a month after induction [43]. It was unknown if genetically predisposed MSCs are always capable of developing into cancers or if it is dependent on the proliferative status of the cell in which a quiescent state could act as a tumor suppressor. Moon et al. demonstrated that activation of mutant Braf and Pten loss did not result in melanocyte expansion or melanomas when MSCs are in a quiescent state, from late-anagen to telogen phase. However, when oncogene activation occurred during early anagen or when the MSCs were artificially stimulated by depilation, there was a large expansion of MSCs and development of melanomas. This was seen by proliferation of *tdTomato* positive cells in the bulge region of the hair follicle and “red” melanomas. Since the induction of *tdTomato* is a permanent and non-reversible event, it is impossible to distinguish differentiation status solely by reporter positivity. It is possible that melanomas arose from a differentiated or transit-amplifying intermediate cell state. However, it is clear from this study that the transition of the follicular MSCs from a non-dividing state to one of active proliferation is a necessary event in melanomagenesis. 

UVB exposure is an extrinsic factor that is also capable of MSC activation and migration to the epidermis with differentiation to Tyrp1^+^ melanocytes. Macroscopic lesions occur around two weeks after UVB exposure and genetic activation, whereas the covered counterparts remain in a quiescent state [25]. The oncogenic hit was required within the MSC compartment, because when 4-hydroxytamoxifen (4-HT) treatment was applied after UVB exposure, and thereby targeting the already migrated and differentiated Tyrp1^+^ melanocytes, there was a stark reduction in melanoma formation [25]. Transcriptomic analysis of depilation and UVB activated MSCs compared to quiescent MSCs showed perturbation in many cellular processes including cell cycle and a significant role of acute inflammation in UVB melanomagenesis. Induction of inflammation by application of 12-*O*-tetradecanoylphorbol-13-acetate (TPA) resulted in epidermal hyperplasia and immune cell recruitment; conversely, the inhibition of inflammation after UVB radiation with an anti-inflammatory steroid dexamethasone reduced MSC migration and induced melanoma formation. While UVB may be activating MSCs directly, it also has an indirect role by inducing the inflammation of the cutaneous microenvironment [25]. A key protein contributing to UVB-mediated activation in a melanocyte-independent manner is the high-mobility group AT-hook 2, Hmga2. Hmga2 is a chromatin-remodeling protein and plays a role as an inducer of epithelial-mesenchymal transition (EMT) in melanoma [44]. However, its role in melanoma initiation has never been investigated. Hmga2 was found to be significantly overexpressed in UVB-exposed tissues, but not within the melanocyte population. When the *Tyr::CreER^T2^*; *LSL-tdTomato*; *Braf^CA/+^*; *Pten^lox/lox^* mice were crossed with *Hmga2^−/−^* mice, there was a reduction in migrating MSCs and melanoma formation after UVB exposure [25]. Additionally, a cytokine screen through an antibody array showed a reduction in the protein expression of six factors, including SDF1/CXCL12, which has been tied to melanoblast migration [45]. Isolation of reporter positive melanoma-susceptible melanocytes from either *Hmga2^+/+^* or *Hmga2^−/−^* mice was equally capable of melanoma formation when transplanted into recipient mice, illustrating that Hmga2 does not function intrinsically within the melanocyte but in the microenvironment. Future studies are necessary to determine the effector cell-type that requires the function of Hmga2, pathways modulated by this protein, how it becomes activated in response to UVB, and its relevance to human melanoma. 

## 7. The Use of *Confetti* Mice to Track Multiple Cell Progenitors

When studying melanomagenesis in the dorsal skin of the mouse, it is important to consider that the skin architecture does not fully resemble what is found in humans, which may affect the clinical relevance of the interpretations. Melanocytes in the human skin reside at the dermal-epidermal junction and within the hair follicle. Frequently, melanomas arise from in situ melanomas, an early stage where growth is restricted to the interfollicular region of the epidermis, suggesting that melanomas may originate from an interfollicular melanocyte population [46]. After the neonatal stage in mice, interfollicular melanocytes can only be found in the tail, which also contains a pigmented epidermis [47]. Considering the dorsal skin of the mouse in isolation may implicate tumor cells of origin that may differ in humans. To study more relevant tissue architecture, studies have investigated the melanomagenic potential of melanocytes residing in the mouse tail with the *R26R-Confetti* mouse. The *Confetti* mice incorporate into the *Rosa26* locus a floxed *Neo^R^*-cassette that acts as a transcriptional roadblock upstream of *Brainbow 2.1 (Br2.1)* [48]. The *Br2.1* construct contains two invertible floxed DNA segments that are placed in tandem to generate three different inversion events, causing differential expression of the four encoded genes: *RFP*, *CFP*, *GFP*, or *YFP* [49]. Homozygous *Confetti* mice label individual cells with one of ten possible color combinations from the stochastic recombination of the fluorophore proteins to distinguish progeny from neighboring stem-cells within a niche. When used in combination with the *Tyr::CreER^T2^* mice, MSCs and their progeny are distinguished from one another [50]. 

The mouse tail epidermis is divided into two distinct regions, the pigmented scale region that is negative for the K10 marker and a non-pigmented inter-scale region that is positive for K10. Using the *Tyr::CreER^T2^*; *Braf^CA/+^*; *Pten^lox/lox^*; and, *R26R-Confetti* quadruple transgenic mouse, Kohler et al. studied the expansion of melanocytes in both scale and interscale compartments of the tail after activation of the melanomagenic program [50]. Pigment-producing melanocytes can be distinguished from amelanotic melanocytes by their expression of a type 1 transmembrane glycoprotein (gp100). Amelanotic MSCs (gp100 negative) were found only in the interscale compartment (K10-positive) and were resistant to melanomagenesis. Through pulse-chase experiments using the iDct-GFP mouse, they showed that these cells maintained a constant GFP signal 9 months after induction. The quiescent state of these amelanotic melanocytes may act as a tumor suppressor and no other perturbations were made that could ‘activate’ these cells, such as UVB exposure. No melanomas appeared to develop from pigment-producing, gp100-positive melanocytes that are located in interscale (K10-positive) region, even though their scale (K10-negative) counterparts expanded rapidly. This suggests that there are microenvironmental factors that contribute to the differences in the melanomagenic potential of different cell states. The growth pattern was characteristic of human melanomas, growing radially about a month after melanoma initiation, followed by invasion into the dermis at about two and a half months [50]. Pigment-producing melanocytes appear to be the cell of origin in this model. It is still possible that amelanotic melanocytes may have migrated and differentiated in the scale region, albeit five days after induction in a time-lapse video of 12 h no interscale labeled cells migrated or moved away, making it unlikely that the amelanotic melanocytes served as the cell of tumor origin [50]. 

Single-cell transcriptomic analysis of FACS-isolated melanocytes and melanomas allowed for the further characterization of these cells. A subset of melanoma cells in the radial growth phase seemed to be differentiated and exhibited a pigment-producing profile, clustering mostly with normal melanocytes and a transcriptome that is driven by Mitf [50]. MITF is one of the major transcription factors in melanocytes and melanoma cells [51]. It regulates phenotype switching and the ability of melanoma cells to adopt a resistant stem-cell like programming [52]. An unpigmented group of radial melanoma cells appeared to be transformed and had a transcriptomic profile for lipid metabolism and EphrinA3 (EFNA3) signaling. This transcriptome cluster was predicted to be dependent on Kruppel-like factor 4 (KLF4), which is a downstream target of the RAS/RAF/MEK/ERK signaling pathway and is required for melanoma survival through the inhibition of apoptosis [53]. There was also a predicted increase in the activity of Slug (Snai2), which is an inducer of EMT and a master regulator of neural-crest specification [54]. Analysis of the two distinct melanomas found in radial growth phase, either driven by Mitf or EphrinA3, determined zinc finger E-box-binding homeobox 1 (Zeb1) to be a driver of the unpigmented state. Zeb1 is a transcription factor and induces EMT in many tumor types [55]. Interestingly, melanomas in the vertical growth phase expressed an invasion and migration signature with many downstream targets of transforming growth factor β (TGF-β) signaling, such as the TEAD transcription factor TEAD4 and the AP1 family transcription factors Jun and Fos. Recent evidence has shown an antagonism between c-Jun and MITF [56]. MITF binds to the c-Jun locus where it negatively regulates its transcription. However, proinflammatory cytokines suppress MITF expression through c-JUN, which leads to a feed forward loop, leading to an increase in c-JUN that results in MITF-low and c-JUN-high cell populations. Consistent with this, the dermal-invading melanoma cells no longer expressed the Mitf transcriptome signature [50]. These data suggest that the loss of differentiation markers and reprogramming to a more neuronal progenitor like state occurs in a subset of melanoma cells before dermal invasion. This switch seems to be triggered by Zeb1 and AP1 transcription factors, but the downstream mechanisms remain unclear. However, this switch could be initiated by an intrinsic mechanism within the melanoma cell where it is in a microenvironment that is starved for nutrients or extrinsic factors involving inflammation and myeloid derived immune cells could be at play. Future studies employing the melanocyte response to UV in the mouse tail will be interesting if the same cells that are found to be susceptible to Braf-driven melanomagenesis are activated by UV. Labeling of the melanocyte compartment in melanomagenic models has begun to tease out intrinsic and extrinsic factors that are important in melanomagenesis. Additional studies identifying and developing specific markers for melanocyte stem cells and their precursors may help in understanding the cell of origin in melanomagenesis [57,58].

## 8. The Fluorescent Ubiquitination-Based Cell Cycle Indicator (FUCCI) Mice

Cell cycle progression and proliferation mark important events in eukaryotic cell biology, specifically during normal development, stem cell renewal, tissue maintenance, and homeostasis. Moreover, any aberrations in the normal cell division cycle may lead to genetic abnormalities, and subsequently, cancer. Therefore, the controlled regulation of cell division cycle is of extreme importance. Most conventional studies involving cell cycle rely on the use of unicellular organisms or immortalized cell lines that are grown in culture. Analysis of cell proliferation using these in vitro approaches involve fixing the sample followed by immunofluorescent staining for cell cycle markers such as nucleotide analogs (BrdU, EdU), or replication proteins (PCNA, Ki-67). Thus, these approaches do not allow for us to monitor cell cycle dynamics in vivo. Moreover, it is largely becoming clearer that fate of a cell with respect to proliferation, dormancy, or exit from cell cycle for differentiation is dependent on its microenvironmental milieu. Thus, to investigate the defects in physiological processes, such as development, regeneration, as well as transformation of normal cell into a malignant phenotype, it is important to first study these processes in a cellular context [59]. 

Recently, a novel reporter technology, named FUCCI (Fluorescent Ubiquitination-based Cell Cycle Indicator), was introduced that enables the monitoring of cell cycle progression in live cells. This technology is based on two proteins that are involved in a DNA replication system of higher eukaryotes, namely, Cdt1 and its inhibitor Geminin. The expression levels of these two proteins are inversely proportional and they fluctuate during cell cycle progression. For instance, Cdt1 levels are the highest in G1 phase and they decline right after the onset of S phase [60]. On the contrary, Geminin levels peak during S and G2 phase, but drop drastically during late mitosis and G1 phase. This technology is referred to as ubiquitination-based because the inverse expression levels of Cdt1 and geminin proteins are regulated by the sequential activation of the E3 ubiquitin ligases APC and SCF. The APC/C ubiquitin ligase, which is functional during G1 phase of cell cycle, targets Geminin for degradation. In contrast, SCF ubiquitin ligase is functional only during S and G2 phases and targets Cdt1 for degradation [61].

The FUCCI system has been extensively used in many organisms to study cell cycle dynamics. Sugiyama et al. generated a FUCCI derivative using zebrafish homologs of Cdt1 and geminin and exploited this cell-cycle dependent proteolysis of two ubiquitination oscillators to study cell-cycle transitions in differentiating notochords of zebrafishes [62]. Zielke et al. developed a drosophila-specific FUCCI system (Fly-FUCCI) that helps to distinguish G1, S, and G2 phases of cell cycle [63]. Fly-FUCCI system incorporates fluorochrome-tagged degrons from the Cyclin B and E2F1 proteins, which are degraded by the ubiquitin E3-ligases APC/C and CRL4Cdt2, during mitosis or the onset of S phase, respectively, and serves as a valuable tool to study cell cycle patterns in various tissues of the drosophila fruit fly [63].

**Figure 2:**
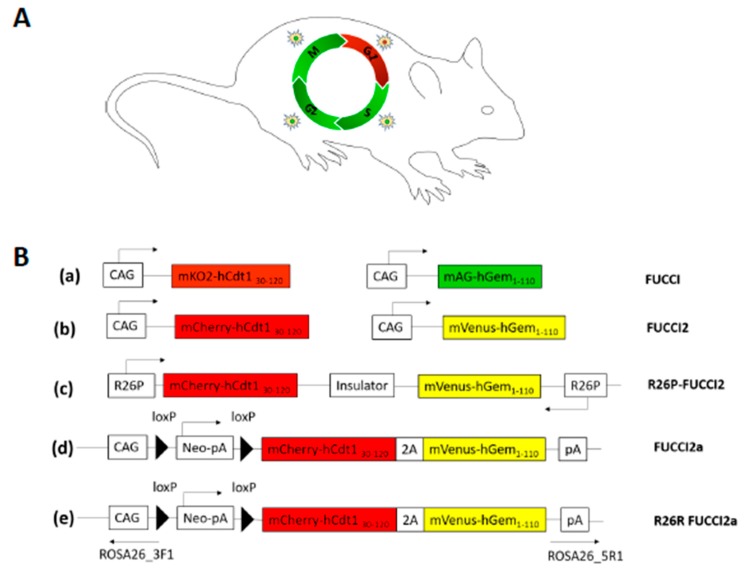
(**A**) The original fluorescent ubiquitination-based cell cycle indicator (FUCCI) mouse model allows labeling of cells in G1 phase with red fluorescence and in G2/M/S phase with Green fluorescence; (**B**): (**a**) The original FUCCI model expression constructs as two separate mouse lines: 1. Expresses fused monomeric Kusabira Orange (mKO2) fluorescent protein with a truncated hCdt1 containing amino acids 30–120 and mark cells residing in G1 phase of cell cycle with red fluorescence. 2. Expresses fused monomeric Azami Green (mAG) fluorescent protein with the 110 amino acid *N*-terminus of the hGeminin protein and mark cells in S/G2/M phase with green fluorescence; (**b**) Another version of the FUCCI called FUCCI2 was developed recently, which replaces the fluorescent proteins mKO2 and mAG with mCherry and mVenus, respectively; (**c**) R26P-FUCCI2 expression construct. mCherry-hCdt130-120 and mVenus-hGem1-110 are bidirectionally expressed from the ubiquitous promoter rosa26 (R26p). This model involved the constitutive labelling of G1 cells with red fluorescence and S/G2/M cells with yellow fluorescence; (**d**) FUCCI2a expression construct in which both the probes are fused together using the Thosea asigna virus 2A (T2A) self-cleaving peptide and involves a bidirectional transgene driving the sensors mCherry-hCdt1 (30/120) and mVenus-hGem (1/110); (**e**) R26R FUCCI2a construct involves incorporating two fluorescent sensors into Rosa26 locus (R26R Fucci2a) conditionally; which allows the cell cycle regulated expression of mCherry (red) in G1 phase and mVenus (green) in S/G2/M phases only upon Cre-recombinase- mediated activation of the transgene.

Sakaue-Sawano et al. generated the FUCCI mouse model system using CAG promoter and is based on fusion of monomeric Kusabira Orange (mKO2) fluorescent protein with a truncated hCdt1 containing amino acids 30–120 and a fusion of monomeric Azami Green (mAG) fluorescent protein with the 110 amino acid *N*-terminus of the hGeminin protein [64]. During the G1 phase of the cell cycle, mKO2-hCdt1 (30/120) probe accumulates inside the cell, which is then degraded by APC/C at the G1-S transition. Similarly, during S/G2/M phases, mAG-hGem (1/110) probe accumulates and is quickly degraded by SCF before cytokinesis. Thus, this powerful system enables the accurate visualization of live cells in either G1 or S/G2/M phase by virtue of which FUCCI probe they express (Figure 2A,B(a)) [64]. Another version of the FUCCI called FUCCI2 was developed recently that replaces the fluorescent proteins mKO2 and mAG with mCherry and mVenus, respectively. These sensors, mCherry that emits red and mVenus that emits green, provide far better color contrast, and can be spectrally separated from GFP (Figure 2B(b)) [65]. 

Despite being a powerful tool of in vivo cell cycle stage visualization, the first generation FUCCI mice displayed several shortfalls. For example, these CAG-FUCCI mice exist as two separate lines CAG-mKO2-hCdt1 (30/120) and CAG-mAG-hGem (1/110), and additional transgenics are usually highly susceptible to transgene inactivation. They also lacked the potential for conditional activation. Variegated or low expression levels were also noticed in certain tissues, which is likely due to random integration of FUCCI probes, leaving CAG promoter inactive or only weakly active in most tissues. Moreover, the corresponding transgenes were located on different chromosomes resulting in difficulties in maintaining the mouse line. To overcome these problems, Abe et al. generated a new transgenic mouse line, R26p-Fucci2 (Figure 2B(c)). This line utilizes the ubiquitously active Rosa26 promoter (R26p) and constitutively expresses both mCherry-hCdt1 (30/120) sensor representing the G1 phase and mVenus-hGem (1/110) sensor representing the S/G(2)/M phase from a single transgene to preserve their co-inheritance [66]. 

To further tackle the above-mentioned problems about the conventional FUCCI mouse models, Mort et al., went on to develop another transgenic mouse line of FUCCI based reporter, called FUCCI2a. FUCCI2a is a bicistronic version in which both the probes are fused together using the Thosea asigna virus 2A (T2A) self-cleaving peptide and involves a bidirectional transgene driving the sensors mCherry-hCdt1 (30/120) and mVenus-hGem (1/110) (Figure 2B(d)) [67]. Moreover, to track cell cycle oscillations in a tissue or cell-type specific manner, they further modified this FUCCI2a transgenic mouse line by incorporating the two fluorescent sensors into the *Rosa26* locus (*R26R Fucci2a*) conditionally, which allowed the cell cycle-regulated expression of mCherry (red) in G1 phase and mVenus (green) in S/G2/M phases only upon Cre recombinase- mediated activation of the transgene (Figure 2B(e)). The construct is generated by placing a floxed STOP cassette in front of each probe. Cre-mediated loxP recombination excises the STOP cassette, resulting in the expression of the FUCCI probes in all Cre-expressing cells [59,68]. 

Although a powerful tool, the FUCCI system displays some shortfalls. FUCCI system does not distinguish G0 and G1 phase of the cell cycle since Cdt1 is expressed in both phases; thereby preventing the proper characterization of cells in quiescent (G0) phase. Recent advances in transgenic mouse technology have given rise to newer FUCCI mouse models. Oki et al. modified the FUCCI model by combining G0-G1 transition reporter mVenus-p27K with FUCCI probes, which enabled identification of quiescent cells and visualization G0-G1 transition [69]. Moreover, conventional FUCCI system also fails to distinguish between the S/G2 and M phase. Since, any of the cell cycle phases may get perturbed during cell cycle abnormalities, a system of simultaneous reporting of all four cell cycle phases would be highly useful. Recently, Bajar et al. developed a reporter of the transition from S to G2 phase by engineering a far-red fluorescent protein, mMaroon1, to visualize chromatin condensation in mitosis. They named this system as FUCCI4, which incorporated a set of four fluorescent indicators to resolve all four cell cycle phases in living cells [70].

A large number mouse models expressing Cre recombinase under the control of a defined promoter have been engineered, which can be crossed with R26R-FUCCI 2 mice to achieve tissue specific expression of FUCCI sensors. For example, in the case of melanocytes, in depth study of their biology in their natural milieu has been very difficult owing to their paucity (only 1%) in mammalian skin. The FUCCI system has proven a valuable tool for this purpose. For instance, Mort et al. crossed *R26Fucci2aR* mice with Tyr::CreB animals expressing Cre recombinase in the neural crest derived melanocyte lineage and utilized this melanocyte-specific expression of FUCCI2a via *Tyr::Cre* mediated activation to study the cell cycle dynamics in 3T3 cells as well as melanoblast cells in mouse embryonic skin samples [67]. Glover et al. further went on to develop another transgenic mouse model (*Rosa26::mT/mG*) in which the *Rosa26* promoter ubiquitously drives the membrane expression of *tdTomato* (red) in all cells, but switches to green membrane fluorescence (EGFP) upon Cre- mediated recombination and the excision of *tdTomato* [47]. In another model of zebrafish, Iyengar et al. utilized FUCCI reporter system to demonstrate that unpigmented *mitfa*-expressing cells directly differentiate into melanocytes during regeneration upon the activation of *Wnt* signaling [71]. Thus, these newer transgenic mouse models that incorporate FUCCI based reporter system are a valuable tool and make fluorescent imaging as well as isolation very convenient in tiny cell populations, such as melanocytes.

## 9. Future Directions

This ability to isolate pure populations of melanocytes allows for detailed investigations into melanoblast and melanocyte development, and basic biology at the cellular and molecular level. Ex Vivo imaging at the single cell level of melanocytes within their environment shows their movement through development, and in response to outside stimuli, such as UVR. Along with melanocyte specific changes, indirect effects from the microenvironment that affect pigment cell function, such as the immune system, cellular interactions with resident keratinocyte and fibroblasts, and tissue architecture can be explored with the described models. Further studies should consider not only transcriptional changes occurring in the pigmented cell population, but also the changes in chromatin-associated proteins, histone modifications, and DNA methylation. While many of the initial studies consider the early development, when considering the melanocyte in an aged microenvironment may be relevant in the understanding of disease phenotypes as age-related epigenetic changes are known to contribute to tumorigenesis [72,73]. The interaction between melanocytes and fibroblasts are critical for melanoma invasion, and therapy resistance [74,75,76,77]. Senescent fibroblasts in the aged microenvironment can promote tumor metastasis through their secretome [78,79,80]. Likewise, UVR can promote melanomagenesis and tumor metastasis in melanoma, and both UVA and UVB wavebands can alter the microenvironment of the skin [2,81,82,83,84]. Further studies on these non-mutational mechanisms of UVR should help to determine its elusive role in tumorigenesis and the respective contribution of each waveband. As mentioned previously, only UVB could cause an interferon-gamma driven immune-response within the skin. It is interesting to note that only UVA requires melanin pigment to induce melanomas in mice [85]. Further studies are needed to determine the interaction between UVR and pigmentation within the melanocyte and the use of an inducible system will facilitate these studies. The use of the FUCCI mouse will allow for the study of quiescent and actively dividing MSCs in these processes. Crossing these mice with developed models of disease will open many new avenues of study. Several mouse models currently are available for studying melanoma, including UVR-inducible models and those that are driven by the most common oncogene found in melanoma, BRAF^V600E^ [1,43,86]. Isolation of melanocytes in these models allows researchers to study any point of disease progression. Further development of these mouse models will help answer more complex questions about melanocyte and melanoma biology.
